# Integrated vector management for malaria control in Uganda: knowledge, perceptions and policy development

**DOI:** 10.1186/1475-2875-11-21

**Published:** 2012-01-14

**Authors:** Clifford M Mutero, Dieter Schlodder, Narcis Kabatereine, Randall Kramer

**Affiliations:** 1 Centre for Sustainable Malaria Control and School of Health Systems and Public Health, University of Pretoria, Private Bag 323, Pretoria 0001, South Africa; 2 Vector Control Division, Ministry of Health, P.O. Box 1661, Kampala, Uganda; 3 Nicholas School of the Environment and Duke Global Health Institute, Duke University, Box 90328, Durham, NC 27708, USA

**Keywords:** Malaria, Integrated vector management, Policy development, Uganda

## Abstract

**Background:**

Integrated vector management (IVM) is increasingly being recommended as an option for sustainable malaria control. However, many malaria-endemic countries lack a policy framework to guide and promote the approach. The objective of the study was to assess knowledge and perceptions in relation to current malaria vector control policy and IVM in Uganda, and to make recommendations for consideration during future development of a specific IVM policy.

**Methods:**

The study used a structured questionnaire to interview 34 individuals working at technical or policy-making levels in health, environment, agriculture and fisheries sectors. Specific questions on IVM focused on the following key elements of the approach: integration of chemical and non-chemical interventions of vector control; evidence-based decision making; inter-sectoral collaboration; capacity building; legislation; advocacy and community mobilization.

**Results:**

All participants were familiar with the term IVM and knew various conventional malaria vector control (MVC) methods. Only 75% thought that Uganda had a MVC policy. Eighty percent (80%) felt there was inter-sectoral collaboration towards IVM, but that it was poor due to financial constraints, difficulties in involving all possible sectors and political differences. The health, environment and agricultural sectors were cited as key areas requiring cooperation in order for IVM to succeed. Sixty-seven percent (67%) of participants responded that communities were actively being involved in MVC, while 48% felt that the use of research results for evidence-based decision making was inadequate or poor. A majority of the participants felt that malaria research in Uganda was rarely used to facilitate policy changes. Suggestions by participants for formulation of specific and effective IVM policy included: revising the MVC policy and IVM-related policies in other sectors into a single, unified IVM policy and, using legislation to enforce IVM in development projects.

**Conclusion:**

Integrated management of malaria vectors in Uganda remains an underdeveloped component of malaria control policy. Cooperation between the health and other sectors needs strengthening and funding for MVC increased in order to develop and effectively implement an appropriate IVM policy. Continuous engagement of communities by government as well as monitoring and evaluation of vector control programmes will be crucial for sustaining IVM in the country.

## Background

Vector control is among the key strategies that are widely promoted by the World Health Organization (WHO) and the Roll Back Malaria Partnership (RBM) for prevention and reduction of malaria [1-3]. The other strategies include early diagnosis and prompt treatment of malaria cases, mainly using artemisnin-based chemotherapies (ACTs), and intermittent preventive treatment in pregnancy [3]. Vector control protects people by preventing, reducing or interrupting the transmission of malaria [1]. There are many different methods of malaria vector control available, including insecticide-treated nets (ITNs), long-lasting ITNs (LLINs) and indoor residual spraying (IRS) [4-7]. While ITNs, LLINs and IRS involve the use of chemical insecticides, some of the other methods of controlling larval or adult mosquitoes apply biological control techniques or environmental management [8,9].

WHO recommends the use of appropriate combinations of non-chemical and chemical methods of malaria vector control in the context of integrated vector management (IVM) [10]. An IVM approach is pragmatic in that it offers a menu of vector control methods which can be applied in various combinations to suit different ecological and socioeconomic settings. Besides, by using a range of different methods, it is possible to effectively target vectors at different stages in their life cycle, for instance, as larvae and pupae in mosquito breeding habitats, or at certain times during the host-seeking and resting behaviour of adult mosquitoes [11]. On the other hand, reliance on only one vector control method is, in the long term, usually unsustainable for a variety of reasons, most notably insecticide resistance and adverse health and environmental impacts in the case of the use of chemical control [8,9].

In 2004, WHO published the "Global Strategic Framework for Integrated Vector Management", spelling out the principles, objectives and requirements of IVM. The document underscores the purpose of IVM as improving the efficacy, cost-effectiveness, ecological soundness and sustainability of vector control [10]. IVM is defined in the document and in a subsequent 'WHO Position Statement on IVM' as "a rational decision-making process for the optimal use of resources for vector control" [12].

This current paradigm of IVM identifies several key elements for successful implementation of the approach [10,13]. They include: integration of non-chemical and chemical vector control methods and their integration with other disease-control measures; evidence-based decision making using methods based on sound knowledge of factors influencing local vector biology, disease transmission and morbidity; capacity building including development of adequate human resources, training and career structures at national and local level to manage IVM programmes; strengthening collaboration within the health sector and with other public and private sectors whose actions and policies might have important implications for vector control; engaging local communities and other stakeholders; and, creating a public health regulatory and legislative framework to reinforce IVM.

**Figure 1:**
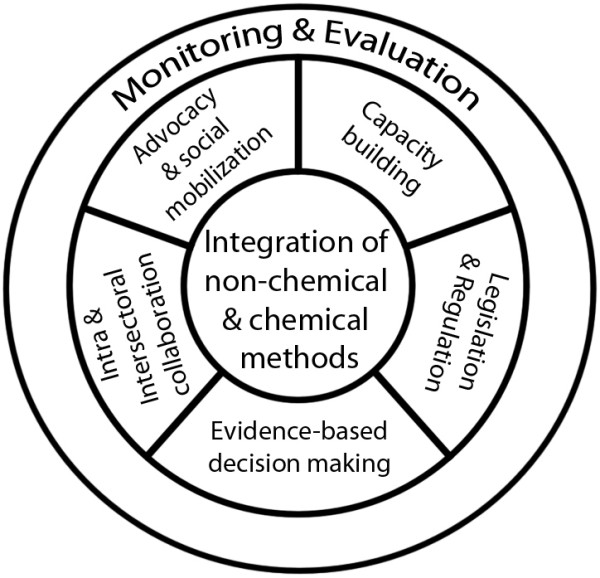
**Diagrammatic representation of integrated vector management (IVM) for malaria control**.

On the basis of the current definition, therefore, IVM for malaria control does not merely focus on how to technically combine different mosquito control methods, as may have been the impression from earlier descriptions and diagrammatic illustration of the concept [14]. Rather, the approach is viewed as also involving programme management aspects implied in the various key elements highlighted above, and without which the integration of mosquito control methods may not be sustainable (Figure 1). However, from an implementation and practical point of view, the present diagrammatic interpretation of IVM for malaria control differs from another, perhaps most recently published, generalized illustration of IVM [13] by visually emphasizing the centrality of the integration of mosquito control methods relative to the other key elements of the approach. Furthermore, monitoring and evaluation is shown in the illustration as being essential for the successful implementation of all the integral components of IVM.

Unfortunately, IVM has not been adopted by national malaria control programmes (NMCPs) of most countries largely due to a lack of country-specific policies to guide the development and implementation of the approach [11,15]. In an effort to overcome such hurdles, WHO has, in collaboration with various stakeholders, continued to explore ways of assisting NMCPs to promote IVM, including the publication of an IVM handbook and guideline documents on IVM and IVM policy development [16,17].

In practical terms, policy development in the health and other sectors usually proceeds in an incremental and iterative manner [18-20]. Accordingly, an IVM policy process would need to take cognizance of and build on existing health sector and other sectors' policies [21]. It also should include consultation with key stakeholders in order to effectively address challenges related to diverse and often competing health, social and environmental objectives [22]. It is in this context that the study was conducted in Uganda. The country does not have a specific and detailed IVM policy framework although it has an overall Malaria Control Strategic Plan that covers various aspects of vector control [15]. Furthermore, Uganda is among countries in Africa with the highest burden of malaria infections and malaria related deaths [23,24].

The objective of the study was to assess knowledge and perceptions in relation to current malaria vector control policy and IVM in Uganda, and to make recommendations for consideration during future formulation of specific IVM policy.

## Methods

The study used qualitative and descriptive methodology. A structured questionnaire was developed and used to interview key stakeholders in November 2010. The respondents were based in Kampala and Entebbe, Uganda's capital and second largest cities, respectively.

### Selection of the study population

Interviews were purposefully conducted among professionals working in health and other sectors whose activities and policies can directly or indirectly impact on malaria vector control [25-28]. The study population comprised of 34 individuals working at senior technical or policy-making level and distributed among the different sectors as follows: Health, 23; Environment, 3; Agriculture, 1; Fisheries, 6; NGO, 1. The majority of the participants worked in government departments including the national malaria control programme (NMCP) while the rest were from universities and NGOs. One participant was from WHO.

### Survey structure and administration

The questionnaire had 21 open-ended and 31 close-ended questions. It was divided into the following four sections, each targeted at a different aspect of malaria:

1. The first section examined general malaria knowledge through questions about knowledge of mosquito ecology, malaria epidemiology and socioeconomic determinants of malaria.

2. The second section assessed the participants' basic knowledge and opinions in relation to malaria vector control (MVC) policy in Uganda, including questions about the deficiencies and successes of MVC policies, as well as how policies could be improved.

3. The third section focused on different methods of MVC, including participants' familiarity with and importance rankings of the various methods currently available.

4. The fourth and final section explored the different key elements of IVM as set out by WHO. These include integration of chemical and non-chemical methods, evidence-based decision making, inter-sectoral collaboration, capacity building, legislation, social mobilization and advocacy.

An informed consent letter was presented to each person participating in the interviews to inform them about the nature of the study and how the information they provided would be used in the final report. All participants accepted the conditions and signed the consent form. Participants were interviewed privately and individually.

### Data handling and analysis

Responses from the interviews were recorded and written down by the interviewer and compiled with the use of Microsoft Excel. The software was also used for data analysis using descriptive statistics.

## Results

### Malaria knowledge

All participants in the study showed good knowledge of malaria when responding to the questions asked to test their basic understanding of the disease. They also indicated that malaria was an important health issue in Uganda. All participants indicated that *Anopheles *mosquitoes were the only known carriers of the malaria parasite. When asked to indicate when malaria was the most serious in Uganda, 85% of participants said it was a few weeks after the rains, coinciding with increased malaria vector populations. The remaining 15% however felt that malaria was a problem throughout all the seasons. These participants reasoned that the climate in Uganda was favourable for mosquito breeding all year round, although peak mosquito populations occurred a few weeks after the rains. This was reinforced by participants stating that Uganda had many wetland areas and that the country was situated on the Equator and thus experienced rains throughout the year. It was also observed that poor drainage of water in towns and rural areas increased the number of mosquito-breeding sites. One participant also mentioned that, due to climate change, the number of areas suitable for mosquito breeding had increased in Uganda, thereby increasing malaria endemicity, which resulted in high infection rates throughout the year.

When participants were asked about the regions they thought were the worst affected by malaria infections, they indicated a wide range of areas. These included northern, eastern, western and south-western areas of Uganda. They specifically named areas such as Apac, Tororo, Kapchorwa, Lango, Oyam, Buraro, Pader and Kiboga. The northern regions of Uganda were specifically mentioned as having the worst incidence of malaria. One participant stated that, due to the environmental conditions in Uganda, prevalence was high throughout the country.

When asked to identify the two population groups that were at the highest risk of serious illness or death due to malaria, the majority of participants selected pregnant mothers and infants under five as the most likely groups, with 48% and 50% respectively. The percentage of responses for the elderly and teenage children was 1% in both cases.

As regards malaria control, 76% of participants felt that a combination of drug therapy and malaria vector control was the best approach of reducing the incidence of malaria infections. Those who considered vector control as being the best approach constituted 21% of the participants while those in favour of drug therapy were 3%. When asked why participants thought that a combination of both methods should be used, many replied that to efficiently reduce malaria infections, one needed to remove the source of infection as well as cure existing infections, and that tackling the malaria problem with all known methods of control would have the greatest impact on reducing infection rates. One participant selected drug therapy alone as the best method, giving the reason that drugs are readily available and that proper vector control is not always implemented. Those supporting vector control as the best approach stated that for Uganda, controlling malaria sources was a simpler option to implement. They also argued that prevention was better than cure and would reduce the burden of curative measures.

Ninety-seven percent (97%) of participants thought that poor socioeconomic status has an impact on increasing the risk of malaria infection of certain population groups. These groups included the impoverished and uneducated rural populations, as well as those in the agricultural sectors. Lack of proper sanitation, inability to afford malaria vector control measures and anti-malarial drugs, limited knowledge of prevention and treatment, poor access to interventions and proper nutrition and the isolation of rural communities were given as some of the reasons. One person stated that: "*as long as Africa remains poor and uneducated, malaria will remain a problem*."

### Malaria vector control policy

All the participants said that they were familiar with the term vector control, although only 75% thought that Uganda had a MVC policy. The majority of participants voiced the opinion that the MVC policy was only somewhat effective. Some of the reasons given for this response were as follows:

• MVC services not reaching all the communities affected by malaria;

• Lack of will and support from government for MVC;

• MVC budget not sufficient to deal with malaria infections properly;

• Neighbouring countries not working together to apply the same methods of control;

• Limited use of various control methods and too many constraints on budget to effectively scale up MVC methods;

• High dependence on outside funding;

• Poor or lack of sanitation;

• Lack of education on use of MVC methods and drug therapies;

• Poor availability of drugs and MVC interventions;

• Current MVC methods are ineffective and were implemented too late;

• Expertise and institutional structures are available, but MVC is not recognized.

Some participants, however, felt that the current approach to MVC was highly effective, citing good results from programmes that have been implemented, and that a combination of ITNs and IRS has been shown to be effective. Those participants that indicated that they thought the approach to MVC was not effective indicated that the different components of MVC are poorly integrated and that interventions do not reach all the affected communities, as well as stating that current methods are ineffective and the policies are not being applied.

Forty-eight percent (48%) of the participants felt that Uganda's approach to MVC was better than that of neighbouring countries with similar malaria situations. The percentage of participants who were unsure how Uganda compared to other countries was 27% while 15% thought the country fared unfavourably and had poorer coverage than surrounding countries. Nine percent (9%) thought that Uganda's approach was the same as that of neighbouring countries. Around a third of people were unsure on how Uganda was fairing in comparison to other countries. The remaining participants who felt that Uganda compared unfavourably or were at the same level said so because Uganda uses different methods of MVC

**Figure 2:**
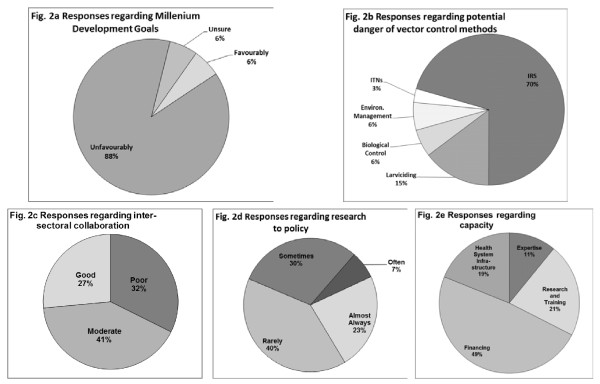
**Participants' responses regarding: a) whether the Millennium Development Goal for Malaria (Target 6 C) will be achieved by 2015; b) the relative potential danger of vector control methods if not managed properly; c) inter-sectoral collaboration in malaria vector control; d) how often research is used to make policy changes; and, e) aspects of capacity and their relative need for strengthening**. (n = 34).

When participants were asked whether they thought the UN Millennium Development Goal (MDG) related to malaria (*Target 6 C: Have halted by 2015 and began to reverse the incidence of malaria and other major diseases*) [29] was achievable, 88% responded unfavourably (Figure 2a). Many respondents reasoned that Uganda's malaria problems were just too big to deal with at current intervention levels, feeling that a massive scale-up in drug and vector control methods would be the only way to achieve the MDG. Budgetary constraints, poor political will, corruption, ineffective interventions and coverage, drug resistance, ignorance and poverty were just some of the obstacles identified by participants as standing in the way of Uganda achieving the MDGs.

### Malaria vector control methods

All persons participating in the study showed good knowledge of the various methods used for MVC. All participants were familiar with IRS, as well as the use of mosquito repellents on one's self. When asked to arrange all the current methods of MVC in order of their perceived effectiveness, participants only ranked those methods with which they were familiar. Counting the number of times a method was placed in a certain position, the list in descending order of perceived effectiveness was as follows: IRS, LLINs, ITNs, environmental management, larviciding, use of mosquito repellents in the house, use of mosquito repellents on one self, biological control.

Participants were also asked to indicate the methods they thought could cause more harm than good if not managed properly. IRS was ranked highest in this regard, followed by larviciding, biological control, environmental management and ITNs (Figure 2b). All participants gave reasons why they thought any of the above mentioned methods may be harmful, indicating some of the following perceived effects for the different methods:

#### IRS

Insecticides, e.g. DDT, are toxic and often inadvertently lead to development of vector resistance; can contaminate food; environmental contamination; people are easily exposed to the toxic chemical; ethical considerations in terms of diseases caused by sprays; poor management can lead to disaster; people don't follow proper guidelines and get overexposed; ecosystem damage can harm industries, e.g. fisheries; can affect biodiversity.

#### ITNs & LLINs

Freshly treated nets may contaminate people, with long lasting effects.

#### Larviciding

Contamination of environment if concentrations incorrect; Incorrect formulations can lead to vector resistance; Fisheries contaminated; Persistent insecticides; Long biodegradation time; Can be ineffective at times; Can contaminate water sources.

#### Biological control

Can contribute to disruption of ecosystems.

#### Environmental management

May affect other species; can create more breeding sites.

### Integrated vector management

When the participants were asked whether they were familiar with the term IVM, all responded that they did, with 45% indicating that they knew the definition of IVM as set out by WHO. All the participants felt that IVM was very important for successful MVC.

When asked whether there was inter-sectoral collaboration in relation to IVM in Uganda, 80% of the participants said that they thought so, but the majority ranked that collaboration as moderate or poor (Figure 2c). Some of the reasons given for the moderate or poor collaboration included: financial constraints; difficulties in involving all possible sectors; political differences; lack of facilities and sensitization; a culture of organizational independence; poor planning and communication; poor report sharing and monitoring and evaluation; lack of commitment.

Sixty-seven percent (67%) of participants responded that communities were actively involved in interventions for malaria control. Participants indicated that when communities are actively engaged by government in implementing mosquito control interventions, they are more accepting of it. Where little is done by government to inform the population about interventions, there is resistance from the communities. Participants pointed out that, the more the effort regarding sensitizing and educating a community, the more readily the community will cooperate in the interventions. Some organizations that participants suggested should become involved in order to strengthen community mobilization included religious institutions, cultural institutions, media, donors, Village Health Teams (VHT) and the private sector.

Forty-eight percent (48%) of the participants felt that the use of research results for evidence-based decision making in vector control interventions in Uganda was inadequate or poor. Thirty-eight percent (38%) thought the use was sufficient while 12% considered it very good. Similarly, the majority felt that malaria research in Uganda is rarely used to facilitate policy changes (Figure 2d). In the case where it was, 45% felt the process was slow, 17% moderately slow, 28% relatively quickly and 10% very quickly.

When asked what Uganda's current capacity for MVC was with regard to expertise, research and training facilities, finances and health system infrastructure, the former two received favourable rankings, with the majority of participants ranking finances and health system infrastructure as below sufficient. Finances were indicated as the aspect of capacity least able to deal with the challenges of IVM and in greatest need of strengthening (Figure 2e). The health, environment and agricultural sectors were given as examples of areas that require the greatest improvement in order for IVM to be better implemented and more effective.

Seventy-five percent (75%) of participants were of the opinion that MVC policies being put in place to support IVM were being adhered to, with all the participants agreeing that such policies were necessary. Some suggestions were made by participants for formulation of specific IVM policy. They included revising any IVM-related policies into a single unified IVM policy and using legislation to improve enforcement among development projects such as those dealing with irrigation and infrastructural development. Making the policies for IVM mandatory for relevant organizations and stakeholders, sensitizing the populace and imposing fines to limit resistance to integrated malaria vector control methods were among suggestions made to improve adherence.

The final question asked participants to rank the different key elements of IVM in order of most to least important. The results were: research for evidence-based decision making; inter-sectoral collaboration; social mobilization and capacity building (both were ranked as equally important); legislation. Participants indicated that they thought the various key-factors were not independent of each other, but were all interlinked and could have profound effects on each other. One participant went as far as suggesting that the key factors were cyclic and that continual evaluation and strengthening were necessary and should be done for IVM to be effective. One of the key factors most mentioned in conjunction with the other factors was legislation.

## Discussion

None of the key elements of IVM [10] was reported at the time of the study as being optimally implemented in MVC in Uganda. For instance, evidence-based decision making was not being commonly used at the programmatic level to select vector control interventions, or in the more general formulation of malaria control policy. Inter-sectoral collaboration and community participation were also perceived as being poor while there was a notable lack of monitoring and evaluation related to MVC. Furthermore, shortage and uncertainty of financial resources as well as a poor health system infrastructure were identified as major impediments to implementation of IVM. The results, however, indicated that the majority of the participants knew the importance of MVC and the need for using an IVM approach. Considering that many participants were from the Ministry of Health, and some even from the National Malaria Control Programme, the disconnect between policymakers' good knowledge of IVM and the lack of implementation of the approach pointed to the need for greater investment in implementation science research related to malaria interventions [30]. The results also confirmed what has previously been reported about the lack of a clear IVM policy in Uganda [15].

Regarding the choice and integration of vector control interventions, the most effective methods were considered to be ITNs/LLNs and IRS. Larviciding and environmental management were regarded as important interventions although not routinely implemented. An area of great concern for participants was the use of DDT for IRS, as evidenced by responses as to which vector control method participants thought had the biggest potential for harm, if not managed properly. DDT has been associated with serious consequences to human health if used indiscriminately for pest control [31], or even when only limited to IRS for malaria vector control [32]. Furthermore, contamination of crops with DDT can have negative economic consequences as it may lead to a rejection of food and other products intended for the export market [33]. Insecticides such as DDT therefore require careful monitoring in order to mitigate unintended negative effects, and also to forestall development of insecticide resistance in malaria vector populations [34].

While the concern about DDT was perhaps an indication of a general awareness in Uganda about the negative health and environmental impacts of chemical pesticides, it warrants further research as it could also have been simply due to political controversy surrounding the use of DDT [24,35]. Conducting prior environmental assessments for vector control interventions and putting in place a monitoring and evaluation system to aid in detection of resistance and informed decision making can have great cost-saving benefits in the long term, as well as ensuring the longevity of public health insecticides currently in use [36].

When asked about the current level of research being done, half of participants felt it was inadequate. However, when it came to research being used to facilitate changes in policy when there was need to adapt current interventions to changing malaria vector or disease situations, participants indicated that such policy changes happened only rarely or sometimes. This slow translation of research into policy changes can result in waste of resources as has been observed in some southern African countries, where interventions that are no longer working effectively are still used [37].

Through responses from participants, it became clear that more needs to be done to monitor effectiveness of interventions. Generally, monitoring and evaluation are necessary in order to improve the efficiency and effectiveness of health interventions and their management, including those for malaria vector control [38,39].

On inter-sectoral collaboration, a few participants commented that there was a lack of shared goals and resources among the various sectors, most notably the health, environment and agricultural sectors. Their recommendation was that the health sector or a special task force should be assigned the role of coordinating IVM between these sectors. Appointing a multidisciplinary team to aid in horizontal communication between different sectors has been shown to be effective in promoting and facilitating inter-sectoral cooperation in public health [40]. It has also been previously recommended for implementation of IVM [39].

Traditionally, legislators have viewed sectors as separate areas, compartmentalizing them and leaving only small areas for cooperation. This has only recently begun to change, with focus moving toward integration [41]. Legislation might thus be an effective way of encouraging cooperation among sectors, while at the same time spelling out the mechanisms and challenges on how it is achieved [42].

Nevertheless, it is important that a sector does not become overly preoccupied with collaborative efforts to a point of being ineffective in performing its core functions. Continual improvement of collaboration through informal networking and formalised and structured planning is seen as one of the best methods of enhancing institutional organisation [40]. One participant emphasized that different sectors should not only share practices, but work to improve communication between them to strengthen cooperation. The agricultural sector as well as the Ministry of Works, which is responsible for sanitation in Uganda, were mentioned specifically by participants as having poor practices that promote mosquito breeding [43]. This is a good example of where not only shared knowledge but also shared resources can promote IVM, especially since the creation of irrigation and sanitation systems falls outside the jurisdiction of the Ministry of Health.

On the basis of the interview results and available literature, Uganda should form multi-sectoral coordinating committees to oversee and facilitate cooperation between the various sectors at the national and district levels [16,44]. Decentralized organizing committees have generally been shown to achieve their designated goals efficiently because of a better focus on local needs of the communities within which they operate [45]. The use of decision analysis support tools can also bring together stakeholders with competing health, environmental and economic objectives to evaluate various options and their trade-offs (22).

Although legislation was placed last when participants were asked to rank the various key elements of IVM, many participants viewed it as a powerful factor for effective implementation of the approach. All participants agreed that legislation was necessary and needed to be improved upon in Uganda. However, using legislation to enforce community participation should perhaps only be considered as a last option, with emphasis rather being placed on educating communities to more willingly improve participation [46]. Generally legislation can play a useful role in guiding the implementation of all the key elements of IVM [47].

As regards MVC-related capacity building needs, the study results corroborated previous observations that Uganda had the necessary entomological expertise to combat malaria and other vector-borne diseases [48]. Unfortunately, such expertise on its own would not be sufficient to overcome the challenges of IVM implementation unless facilitated by well defined career pathways for vector specialists and an appropriate vector control infrastructure. Generally, the following four types of capacities are needed in a systems-based approach to health research and development [49,50]: human capacity, i.e. individual skills and creativity; physical capacity, i.e. laboratories and equipment; organizational capacity, including management, strategies and decision-making capabilities; and social and governmental capacity, i.e. the requisite financial, social and political support for research. Addressing deficiencies in all the four areas of capacity would be necessary for the successful implementation of MVC for malaria control in Uganda. It is worth noting that the lack of funds as cited in the present study reinforces the need for strengthening IVM's key element on inter-sectoral collaboration in order to share not only technical expertise but also the financial cost of sustaining MVC.

While the above factors mainly relate to institutional capacity building, it would be equally important to keep malaria knowledge among communities in Uganda at a high level. The problem of illiteracy, particularly in rural settings, was mentioned by participants as creating high risk population groups that are affected the most by malaria. Studies elsewhere have shown that as malaria in a community decreases, so does the awareness of the dangers of the disease, which could in turn lead to an increase in the incidence of cases due to failure to observe the necessary measures [51].

Regarding monitoring and evaluation, it is recommended that Uganda should train teams at the village level to undertake basic monitoring of MVC among communities, as was done in the past as part of the country's efforts to reduce HIV/AIDS [52]. Timely reporting of information has been found in neighbouring Tanzania as aiding malaria control programme managers in the prompt identification of problems that arise during implementation of interventions [53]. Such village-level feedback could be crucial in helping programme managers in Uganda to assess whether or not particular IVM initiatives are successful.

Finally, it was obvious from the results that IVM policy development in Uganda would have to take into consideration the prevailing political and socio-economic context to ensure implementation. Both poverty and political agenda were viewed as constituting critical barriers to implementation of currently available vector control methods. Interest in understanding the political and social dimensions of policy making in sectors which traditionally relied on having only compelling scientific evidence in order to institute any policy changes has been growing. The trend has in recent years been discussed in detail for policy processes in general [19], and in development of healthy public policy in particular [18].

## Conclusion

Integrated management of malaria vectors in Uganda remains an underdeveloped component of malaria control policy. Cooperation between the health and other sectors needs strengthening in order for the country to be able to develop and effectively implement an appropriate IVM policy. Continuous engagement of communities by government as well as monitoring and evaluation of vector control programmes will be crucial for sustaining IVM in the country. Further, simultaneous research on the key elements of IVM will also be necessary to help overcome the technical, policy and community-participation challenges reported in the study, and which normally hinder implementation of the approach in Uganda and other countries [11,13,15].

## Competing interests

The authors declare that they have no competing interests.

## Authors' contributions

CMM conceptualized the study and drafted the manuscript with DS. DS conducted the interviews and interpreted the results with CMM. All the authors contributed to formulation of the interview questions. All authors have read and approved the final version of the manuscript.

## References

[B1] WHO Malaria vector control and personal protection: report of a WHO study group. WHO technical report series 936 2006 Geneva: World Health Organization 16623084

[B2] WHO A global strategy for malaria control 1993 Geneva: World Health Organization

[B3] RBM Key facts, figures and strategies: the Global Malaria Action Plan 2008 Roll Back Malaria Partnership

[B4] Okumu FO Moore SJ Combining indoor residual spraying and insecticide-treated nets for malaria control in Africa: a review of possible outcomes and an outline of suggestions for the future Malar J 2011 10 208 10.1186/1475-2875-10-208 21798053 PMC3155911

[B5] Lengeler C Insecticide-treated bed nets and curtains for preventing malaria Cochrane Database Syst Rev 2004 2 CD000363 15106149 10.1002/14651858.CD000363.pub2

[B6] Pluess B Tanser FC Lengeler C Sharp B Indoor residual spraying for preventing malaria Cochrane Database Syst Rev 2010 4 CD006657 20393950 10.1002/14651858.CD006657.pub2 PMC6532743

[B7] WHO Global Malaria Action Plan 2009 World Health Organization

[B8] Rozendaal JA Vector control: Methods for use by individuals and communities 1997 World Health Organization

[B9] WHO Vector control for malaria and other mosquito-borne diseases. Report of a WHO Study Group. WHO Technical Report Series 857 1995 World Health Organization 8540245

[B10] WHO Global strategic framework for integrated vector management 2004 World Health Organization

[B11] Townson H Nathan MB Zaim M Guillet P Manga L Bos R Kindhauser M Exploiting the potential of vector control for disease prevention Bull World Health Organ 2005 83 942 947 16462987 PMC2626501

[B12] WHO WHO position statement on integrated vector management 2008 World Health Organization

[B13] Beier JC Keating J Githure JI Macdonald MB Impoinvil DE Novak RJ Integrated vector management for malaria control Malar J 2008 7 Suppl 1 S4 10.1186/1475-2875-7-S1-S4 19091038 PMC2604879

[B14] WHO Manual on environmental management for mosquito control: With special emphasis on malaria vectors. WHO offset publication. No. 66 1982 World Health Organization 6129760

[B15] Biscoe M Mutero CM Kramer R Current policy and status of DDT use for malaria control in Ethiopia, Uganda, Kenya and South Africa. Working Paper 95 2005 Colombo: International Water Management Institute, IWMI

[B16] WHO Handbook in integrated vector management (IVM). Final Draft 2010 Geneva: World Health Organization Available online: http://www.rbm.who.int/partnership/wg/wg_itn/docs/ws8/IVMhandbook.pdf.

[B17] Henk HVN Mutero CM Ichimori K Guidance on policy development for integrated vector management (IVM) 2012 World Health Organization in press

[B18] Fafard P Evidence and healthy public policy: Insights from health and political sciences 2008 National Collaborating Centre for Healthy Public Policy

[B19] Keely JE Influencing policy processes for sustainable livelihoods: Strategies for change. Lessons for change in policy & organizations No.2 2001 Brighton: Institute of Policy Development Studies

[B20] Pasteur K Policy processes: what are they, and how can they be influenced in support of sustainable livelihoods? 2001 Brighton: Institute of Development Studies

[B21] WHO Guidelines for integrated vector management 2003 Harare: World Health Organization Regional Office for Africa

[B22] Kramer R Dickinson KL Johnson RW Anderson RM Fowler VG Miranda ML Mutero CM Saterson KA Wiener JB Using decision analysis to improve malaria control policy making Health Policy 2009 92 133 140 10.1016/j.healthpol.2009.02.011 19356821 PMC3645449

[B23] Gallup JL Sachs JD The economic burden of malaria Am J Trop Med Hyg 2001 64 1_suppl 85 96 11425181 10.4269/ajtmh.2001.64.85

[B24] Muganga G Malaria control for rural areas in Uganda: localizing the interventions MWJ 2011 2 2 Available online: http://www.malariaworld.org/sites/default/files/MWJ%202011%202_2.pdf 10.5281/zenodo.10998617 PMC11145564 38836129

[B25] Hoek WD Malaria and agriculture. (Special issue: Malaria and Agriculture) Acta Trop 2004 89 95 259 10.1016/j.actatropica.2003.10.013 14732232

[B26] Keiser J Castro MCD Maltese MF Bos R Tanner M Singer BH Utzinger J Effect of irrigation and large dams on the burden of malaria on a global and regional scale Am J Trop Med Hyg 2005 72 392 406 15827275

[B27] Birley MH The health impact of development projects 1995 UK: HMSO

[B28] WHO Health and environment in sustainable development 1997 Geneva: World Health Organization

[B29] WHO Global health observatory, health related Millennium Development Goals 2010 World Health Organization Available online: http://www.who.int/gho/mdg/goals_targets/en/index.html

[B30] Sanders D Haines A Implementation research is needed to achieve international health goals Plos Med 2006 3 719 722 10.1371/journal.pmed.0030186 PMC1472551 16729844

[B31] WWF Resolving the DDT dilemma: protecting biodiversity and human health 1998 Toronto and Washington: World Wildlife Fund

[B32] Bornman MS de Jager C Worku Z Farias P Reif S DDT and urigenital malformations in newborn boys in a malarial area BJU Int 2010 106 405 411 10.1111/j.1464-410X.2009.09003.x 19849691

[B33] Morner J Bos R Fredrix M Reducing and eliminating the use of persistent organic pesticides: guidance on alternative strategies for sustainable pest and vector management 2002 Geneva: UNEP/FAO/WHO

[B34] Yewhalaw D Wassie F Steurbaut W Spanoghe P Van Bortel W Denis L Tessema DA Getachew Y Coosmans M Duchateau L Speybroeck N Multiple insecticide resistance: an impediment to insecticide-based malaria vector control program PLoS ONE 2011 6 e16066 10.1371/journal.pone.0016066 21264325 PMC3020220

[B35] Okia M IRS communication strategy: Uganda experience Pan African Malaria Vector Control Conference 2009 Compendium, Research Triangle Park, NC 2009 RTI International Available online: http://www.rti.org/pubs/malaria_vector_control_report_2009.pdf

[B36] USAID Integrated vector management programs for malaria vector control: Programmatic environmental assessment. Update draft 2011 Bureau for Global Health. United States Agency for International Development

[B37] Cliff J Lewin S Woelk G Fernandes B Mariano A Sevene E Daniels K Matinhure S Oxman A Lavis J Policy development in malaria vector management in Mozambique, South Africa and Zimbabwe Health Policy Plan 2010 25 372 383 10.1093/heapol/czq008 20176574 PMC3072826

[B38] Chataway J Chaturvedi K Hanlin R Mugwagwa J Smith J Wield D Kalua F, Awotedu A, Kamwanja L, Saka J Building the case for systems of health innovation in Africa Science, technology and innovation for public health in Africa 2009 New Partnership for Africa's Development (NEPAD) 7 52

[B39] Manga L Toure A Shililu J Implementation of integrated vector management in the WHO-African Region: progress report 2000-2003 2004 Washington: Environmental Health Project

[B40] Delaney FG Muddling through the middle ground: theoretical concerns in intersectoral collaboration and health promotion Health Promot Int 1994 9 217 225 10.1093/heapro/9.3.217

[B41] Axelsson R Bihari-Axelsson S Integration and collaboration in public health--a conceptual framework Int J Health Plann Manage 2006 21 75 88 10.1002/hpm.826 16604850

[B42] Boo CS Legislation for control of dengue in Singapore Dengue Bulletin 2001 25 69 73

[B43] Mabaso MLH Craig M Ross A Smith T Environmental predictors of the seasonality of malaria transmission in Africa: the challenge Am J Trop Med Hyg 2007 76 33 38 17255225

[B44] Hurley J Birch S Eyles J Geographically-decentralized planning and management in health care: some informational issues and their implications for efficiency Soc Sci Med 1995 41 3 11 10.1016/0277-9536(94)00283-Y 7667671

[B45] Casman EA Dowlatabadi H The contextual determinants of malaria 2002 Washington: Resources for the Future

[B46] Chadee DD Williams FLR Kitron ED Impact of vector control on a dengue fever outbreak in Trinidad, West Indies, in 1998 Trop Med Int Health 2005 10 748 754 10.1111/j.1365-3156.2005.01449.x 16045461

[B47] WHO Vector-borne diseases: addressing a re-emerging public health problem 2005 World Health Organization, Regional Committee for the Eastern Mediterranean EM/RC52/3

[B48] Mutero CM Directory of African institutions with existing capacity for training in integrated vector management (IVM) 2010 RTI-International/USAID support for capacity-building in integrated vector management (IVM Task Order II) Available online: http://www.rbm.who.int/partnership/wg/wg_itn/docs/ws8/DirectoryIVMinstitutionsAfrica.pdf

[B49] Csaszar M Lal B Improving health in developing countries Issues in Science and Technology 2006 University of Texas at Dallas

[B50] Roper WL Baker EL Jr Dyal WW Nicola RM Strengthening the public health system Public Health Rep 1992 107 609 615 1454972 PMC1403710

[B51] Atkinson JAM Fitzgerald L Toaliu H Taleo G Tynan A Whittaker M Riley I Vallely A Community participation for malaria elimination in Tafea Province, Vanuatu: part I. Maintaining motivation for prevention practices in the context of disappearing disease Malar J 2010 9 93 10.1186/1475-2875-9-93 20380748 PMC2873527

[B52] Komakech I The village health team strategy is a "most innovative community practice" award winner: the experience of a village volunteer program in Yumbe District, Uganda Health Policy Dev 2007 5 21 27

[B53] Hanson K Nathan R Marchant T Mponda H Jones C Bruce J Stephen G Vouchers for scaling up insecticide-treated nets in Tanzania: methods for monitoring and evaluation of a national health system intervention BMC Public Health 2008 8 205 10.1186/1471-2458-8-205 18544162 PMC2442068

